# Application of Sparse Representation in Bioinformatics

**DOI:** 10.3389/fgene.2021.810875

**Published:** 2021-12-15

**Authors:** Shuguang Han, Ning Wang, Yuxin Guo, Furong Tang, Lei Xu, Ying Ju, Lei Shi

**Affiliations:** ^1^ Yangtze Delta Region Institute (Quzhou), University of Electronic Science and Technology of China, Quzhou, China; ^2^ Beidahuang Industry Group General Hospital, Harbin, China; ^3^ School of Mathematics and Statistics, Hainan Normal University, Haikou, China; ^4^ School of Electronic and Communication Engineering, Shenzhen Polytechnic, Shenzhen, China; ^5^ School of Informatics, Xiamen University, Xiamen, China; ^6^ Department of Spine Surgery, Changzheng Hospital, Naval Medical University, Shanghai, China

**Keywords:** sparse representation, gene expression profile, machine learning, low-rank representation, cancer

## Abstract

Inspired by L1-norm minimization methods, such as basis pursuit, compressed sensing, and Lasso feature selection, in recent years, sparse representation shows up as a novel and potent data processing method and displays powerful superiority. Researchers have not only extended the sparse representation of a signal to image presentation, but also applied the sparsity of vectors to that of matrices. Moreover, sparse representation has been applied to pattern recognition with good results. Because of its multiple advantages, such as insensitivity to noise, strong robustness, less sensitivity to selected features, and no “overfitting” phenomenon, the application of sparse representation in bioinformatics should be studied further. This article reviews the development of sparse representation, and explains its applications in bioinformatics, namely the use of low-rank representation matrices to identify and study cancer molecules, low-rank sparse representations to analyze and process gene expression profiles, and an introduction to related cancers and gene expression profile database.

## Introduction

In recent years, inspired by L1-norm minimization methods, such as basis pursuit (Donoho and Huo, 2001), compressed sensing (Candes et al., 2004; Candes and Tao, 2005; Lustig et al., 2007), and Lasso feature selection (Tibshirani, 1996), sparse representation shows up as a novel and potent data processing method. Sparse representation has been applied to pattern recognition, for example, digit recognition, speech recognition, and face recognition, and achieved good results. Hang and Wu (2009) first introduced sparse representation to the analysis of tumor gene expression data. They applied sparse representation to classify two multi-class tumor data, compared them with the classification performance of a support vector machine (SVM), and concluded that sparse representation was superior to SVM. Sparse representation was subsequently adopted for feature selection and the classification of tumor gene expression data. Hang applied it to gene selection and obtained sound classification results (Hang, 2009). Zheng et al. (Gan et al., 2013) proposed a sparse representation classification method based on meta-samples. The method uses singular value decomposition to extract the meta-samples of various training samples, and then uses the meta-samples to linearly represent test samples and categorizes them based on representation coefficients. The test samples compare the classification performance of this method with other classic methods on multiple two-class and multi-class datasets. The experimental results demonstrated that this method is superior to a classic SVM and other methods. These results testify the application potential of sparse representation methods in tumor gene expression data analysis.

The low-rank sparse representation model based on sparse representation has also become a topic of great interest in fields such as machine vision, machine learning, and image processing, and has been applied successfully in video image processing, target recognition, task learning, and recommendation systems (Huang et al., 2017; Yu and Gao, 2019; Liu et al., 2020a; Yu et al., 2020). In low-rank sparse representation theory, a noisy or missing data matrix is decomposed into an accurate data matrix and a singular/sparse data matrix, where the accurate data matrix has low-rank characteristics, and the singular/sparse data matrix contains data noise and singular data (Tang et al., 2020). Wright et al. proposed a classification algorithm based on sparse representation (Wright et al., 2009a) that successfully applies sparse representation theory to face recognition. Meanwhile, researchers have applied the sparsity of vectors to that of matrices, and proposed low-rank matrix recovery theory (Wright et al., 2009b; Emmanuel et al., 2009) and matrix low-rank representation (Liu et al., 2010). Low-rank representation has also received extensive attention from researchers and has become another important data representation method. It has demonstrated great potential. Sparse representation has many advantages, such as insensitivity to noise, strong robustness, insensitivity to selected features, and no “overfitting” phenomenon. Therefore, the application of sparse representation in bioinformatics should be studied further.

In recent years, inspired by discriminant analysis, researchers have combined discriminative ideas with sparse representation or low-rank representation theory to extract discriminative information from samples further to improve recognition performance. Discriminant analysis is a multivariate statistical analysis method that analyzes various characteristic values of sample data, and then discriminates the category of the observed sample. For example, Fisher Linear Discrimination (FLD). The essence of the FLD is to project sample points into a low-dimensional space so that, in the projected space, the distance between sample points of the same category is small and the distance between sample points of varying categories is large.

And because gene expression profile data research plays a vital role in genetic engineering, protein design, new drug development, etc., the use of machine learning methods including deep learning to explore gene expression profile data modeling methods has led to the biological field Wide attention of researchers. At the same time, the innovation of this article are; 1) The low-rank representation (LRR) is modified, and a new type of low-rank representation model is constructed by introducing manifold regularization and class label restriction mechanism, which is used for low-rank scoring of gene features and selecting the optimal gene subset; 2) Introduce the idea of deep learning to the low-rank sparse model, and propose a deep feature representation method for gene expression profile data, and realize the classification and clustering of gene data on this basis; 3) Propose a feature selection mechanism for gene expression profile data based on low-rank graphs; 4) Establish a genetic feature correlation measurement criterion based on low-rank representation coefficients, use this criterion to obtain a new genetic feature selection method, and use Robust Principal Component Analysis (RPCA) and Maximum Interval Criterion (MMC) to build a two-step genetic feature selection method.

## Database for the Applied Research of Sparse Representation

As sparse representation and low-rank representation have been widely applied to the analysis and research of cancer and gene expression profiles in recent years, the databases of cancer and gene expression profiles can be adopted, respectively, for the research and application of sparse representation methods. Tables 1, 2 show the specific database description.

**TABLE 1:** Common cancer databases.

Database name	Database introduction
GEO Edgar et al. (2008)	The GEO database stores the records (series, samples, and platforms) provided by the original submitter and the sorted data set, but not all the records provided by the original submitter have been assembled into a selected data set. And the selected data sets form the basis of GEO’s advanced data display and analysis functions
TCGA Tomczak et al. (2015)	The Cancer Genome Atlas (TCGA) is a publicly funded project aimed at cataloging and discovering major oncogenic genome changes in order to create a comprehensive “atlas” of cancer genome maps. So far, TCGA researchers have passed large-scale genome sequencing and synthesis Multidimensional analysis analyzed a large cohort of more than 30 human tumors
KEGG Rédei. (2012)	The Kyoto Encyclopedia of Genes and Genomes (KEGG) is a knowledge base for analyzing gene function based on genetic and molecular network systems. KEGG maintains the GENES database and the LIGAND database
COSMIC Forbes et al. (2011)	COSMIC provides comprehensive information about somatic mutations in human cancers. Version v48 (July 2010) describes more than 136,000 coding mutations in nearly 542,000 tumor samples; it aims to collect, manage, organize and present cancer somatic mutations in the world. The information is provided free of charge in a variety of useful ways and can be accessed at http://www.sanger.ac.uk/cosmic
UCSC Cancer Genomics Browser	UCSC Cancer Genomics Browser is a set of web-based tools designed to integrate, visualize and analyze genomic and clinical data. It consists of three main components: hgHeatmap, hgFeatureSorter and hgPathSorter, which can be browsed at https://cancer.cse.ucsc.edu/. And because UCSC Cancer Genomics Browser is an extension of UCSC Genome Browser; therefore, it inherits and integrates the rich human biology and genetics data set of Genome Browser to enhance the interpretability of cancer genomics data
ArrayMapCancer	ArrayMap provides preprocessed tumor genome chip data and CNA maps. In the ArrayMap database, users can search for samples they are interested in, and on this basis, analyze the CNA on the gene or genome fragment of interest

**TABLE 2:** Commonly used gene expression profile database.

Name database	The data source	Database introduction
RNA-Seq Atlas	Network-based RNA-Seq gene expression profile and query tool library	This is the first open-access database that provides data mining tools and large-scale RNA-Seq expression profiling. Its application will be multifaceted, because it will help to identify tissue-specific genes and expression profiles, compare gene expression profiles between different tissues, and systems biology methods that link tissue function to changes in gene expression
GEO	The National Center for Biotechnology Information (NCBI) was established	The initial goal was to serve as a public repository for high-throughput gene expression data mainly generated by microarray technology. In addition, the database also includes comparative genome analysis, chromatin immunoprecipitation analysis describing genomic protein interactions, non-coding RNA analysis, SNP genotyping, and genome methylation status analysis
ArrayExpress	Alvis Brazma from EBI et al	It is a functional genomics database under the European Bioinformatics Association (EMBL-EBI), which collects and organizes data from genomics experiments based on microarrays and sequencing to support reproducible research. It is also one of the main knowledge bases for functional genomics experiments based on microarray and high-throughput sequencing. All data is provided in MAGE-TAB format

## Application of Sparse Representation in Bioinformatics

The development of bioinformatics is mainly divided into three stages: gene stage, genomic stage, and post-genomic stage. The first two stages mainly focus on the research of gene sequences (Yu et al., 2019; Cai et al., 2020a; Fu et al., 2020; Wang et al., 2020; Dao et al., 2021a; Dao et al., 2021b; Huang et al., 2021). In the post-genome stage, bioinformatics has entered a new development period, and its research focus has shifted from the study of gene sequences to the study of gene functions (Wang et al., 2013; Dong et al., 2020; Wang et al., 2021a; Lv et al., 2021; Yu et al., 2021). It incorporates all aspects of the process of acquiring, storing, processing, distributing, and explaining biological information, and combines various tools of applied mathematics, computer science, and biology to clarify and understand biological significance in biological data.

### Cancer Molecular Study Based on Low-Rank Representation Learning

**FIGURE 1:**
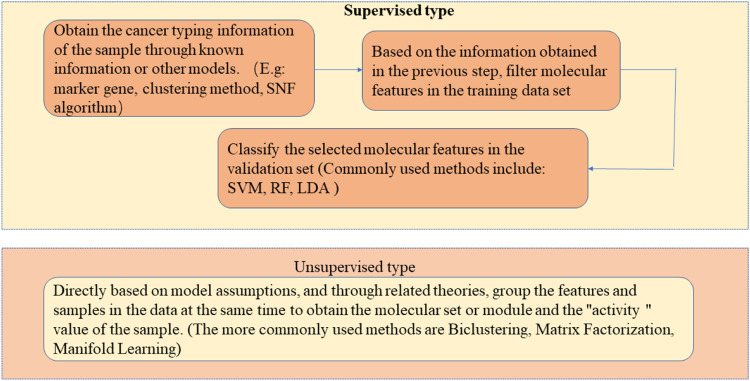
Method for mining cancer molecular features.

**FIGURE 2:**
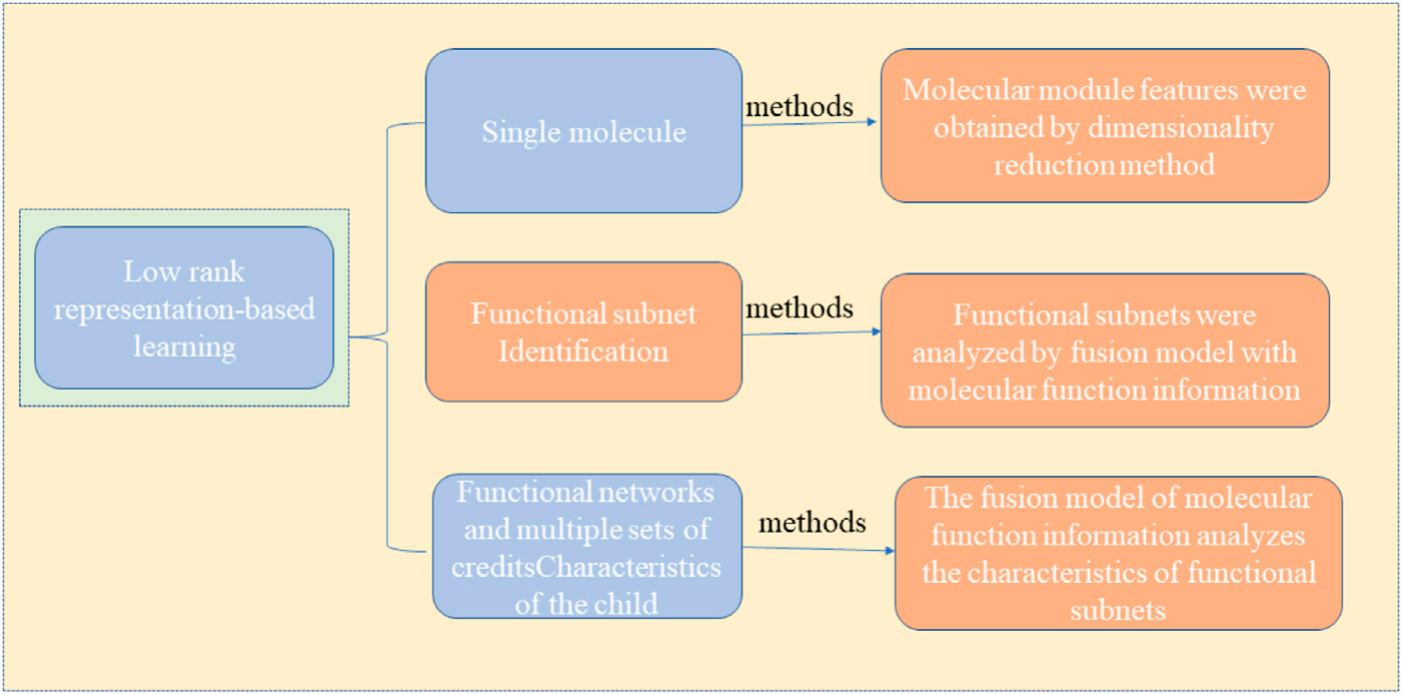
Method for mining cancer molecular features using a low-rank representation matrix.

As a common malignant tumor, cancer is a common fatal disease worldwide because of its complex pathogenic factors, high treatment difficulty, and high risk of recurrence and metastasis. In China, deaths from cancer are always high, and it is a severe threat to the lives and health of Chinese people (Silverberg and Lubera, 1998; Chen et al., 2020). How to prevent and treat cancer effectively has become a topic of widespread concern the world over. With the development of high-throughput sequencing technology, scientists can observe the gene expression of cancer cells at the single-cell level. Feature mining methods for cancer molecules are divided into supervised and unsupervised learning, as shown in Figure 1. The supervised method generally includes two steps: 1) First obtain the cancer classification information of the research sample through known prior information or other models. For example, using marker genes, clustering methods, or SNF algorithms. 2) Based on the sample typing information obtained in the previous step, the candidate molecular characteristics are screened out in the training data set, and then these candidate molecular characteristics are classified or survival analysis in the validation data set to determine the final effective molecular characteristics. The methods often used in this step mainly include difference hypothesis testing, support vector machine algorithm, random forest and linear discriminant analysis. Another type of unsupervised method does not require the typing information of a given sample set. It is mainly based on model assumptions and related data theories. At the same time, the molecular features and samples in the data are grouped to obtain a molecular set or module, and for the “liveness” value of the sample in the new feature space, commonly used methods include bi-clustering algorithm, matrix decomposition and manifold learning. However, existing unsupervised methods (Chen et al., 2020; Zou et al., 2020) fail to distinguish different feature subspaces. Hence, they may produce errors, or even invalid results, when applied to cancer molecular feature mining. Thus, a low-rank representation learning algorithm (Chen and Yanga, 2014) is presented based on the presumption that the sample subspace exists, and samples in the same subspace can represent each other, while those in different subspaces cannot. The algorithm can accurately identify a “clustered” structure or grouping information of inherent samples in the heterogeneous data. The effectiveness of this method has been widely recognized in image processing, and it also provides new ideas and directions for establishing accurate models for mining cancer molecular characteristics. Therefore, a mathematical model based on low-rank representation can be established by combining multiple scales, including molecules, modules, functional networks, and multi-omics molecular features. This model can be studied from the three aspects described below, and a series of mathematical models that are more in line with the heterogeneous structure of data and the biological characteristics of the disease are proposed, and a fair evaluation of the validity and practicability of the model is provided using simulated cases and the application of real data, and theoretical modeling and tools for analyzing multi-scale molecular characteristics of cancer are provided. Figure 2 shows the method of applying a low-rank representation matrix to mine the molecular characteristics of cancer.

1) A dimensionality reduction method is adopted to obtain the characteristics of the molecular module specific to the cancer subtype (Cheng et al., 2018; Tang et al., 2018; Yu et al., 2018; Zhang et al., 2018; Jiang et al., 2019; Su et al., 2019; Liu et al., 2020b; Su et al., 2020). It can address nonlinear sample structure issues that the traditional dimensionality reduction method cannot identify. This is because the dimensionality reduction model fused with low-rank representation learning can process highly heterogeneous data, adaptively capture sample cluster structure and subtype-specific module features, and improve the ability to classify tumor subtypes and obtain reliable molecular modules.
2) The fusion model with molecular function information was used to analyze the characteristics of functional subnets. Makes full use of the advantages of known functional information in biological interpretability (Liu et al., 2019; Cai et al., 2020b), deeply probes into functionally abnormal biological pathways or molecular behaviors, obtains subtype-specific functional subnets, and clarifies the molecular mechanism of cancer from a functional level.
3) A fusion model with molecular function information analyzes the features of functional subnets, makes full use of the biological characteristics of the sample representation relationship consistency of multi-omics data, further explores synergistic or complementary molecular characteristic information at the system level, and provides new clue to enable the understanding of the cross-omics pathogenic factors of cancers.

At the same time, medical imaging is also playing an increasingly major role in helping doctors to conduct a precise diagnosis of cancer. Even medical imaging cloud and remote image center can be used for cloud reading, remote consultation, health management, disease diagnosis, image archiving and communication, etc. (Mehto and Mehra, 2016; Ma et al., 2020; Meziane, 2020; Zhang et al., 2021). Therefore, how to protect patients’ personal information in medical images, such as CT, MRI, and other medical images, so that this personal information and patients’ electronic medical records cannot be leaked has constituted a key issue for the medical industry that needs to be resolved urgently against the background of machine learning cloud computing and big data. Using medical image digital watermarking technology is an effective method to work out this problem (Hong et al, 2016; Vairaprakash and Shenbagavalli, 2017; Shen et al., 2018; Yang et al., 2018; Zhou et al., 2020). Compared with general digital watermarking technology, digital watermarking technology used in medical images, theoretically, should satisfy three characteristics: reliability, availability, and confidentiality.

**FIGURE 3:**
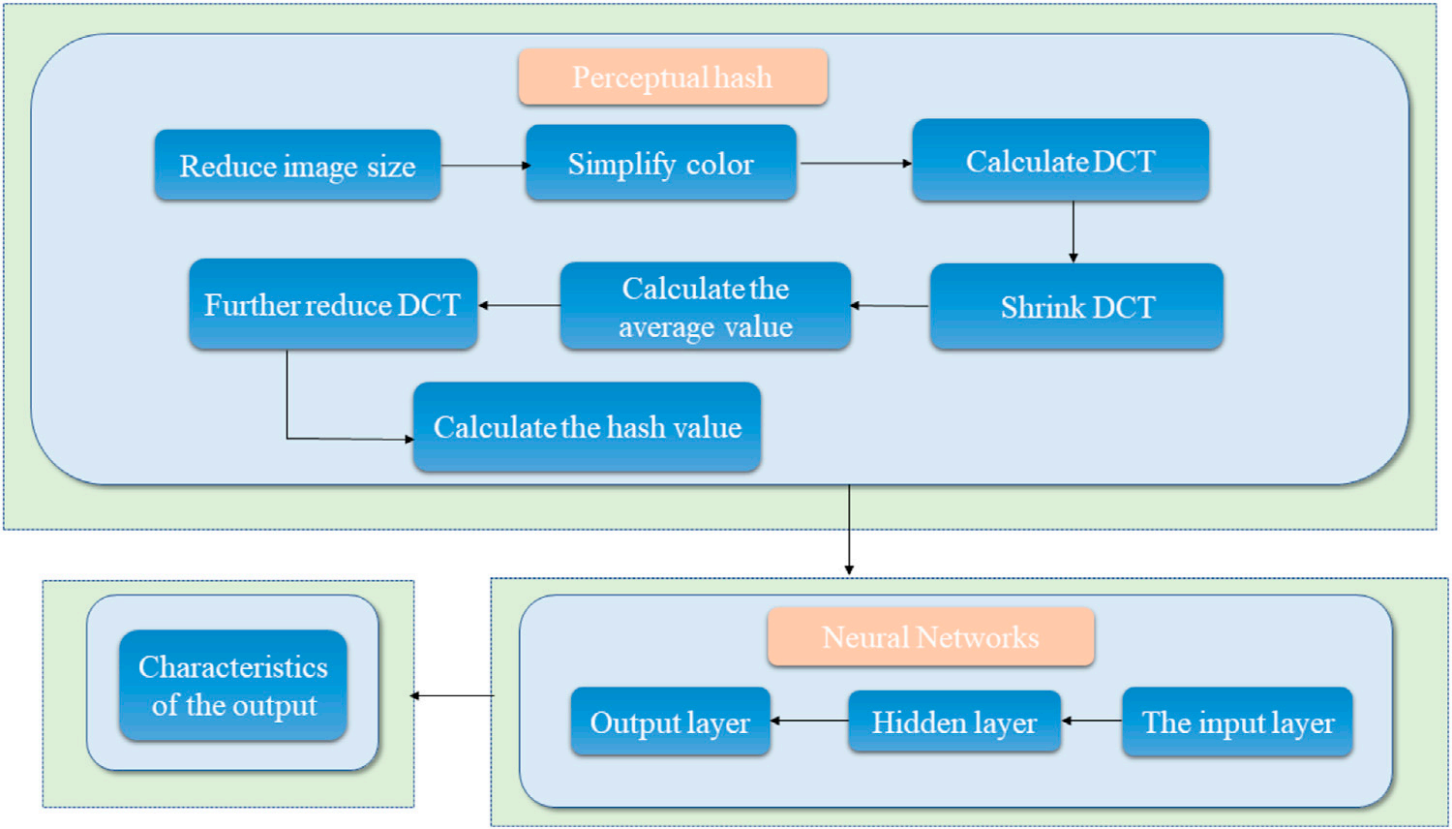
Schematic diagram of the robustness feature acquisition of medical images based on perceptual hashing and a neural network.

Common medical image digital watermarking algorithms are divided into three categories: 1) a medical image watermarking algorithm based on non-interest area RONI (Thanki et al., 2017), which diminishes the watermark embedding capacity (Liu et al., 2016b; Gangadhar et al., 2018) and demonstrates poor robustness; 2) reversible digital watermarking; 3) classic conventional digital watermarking algorithms used to process medical images. However, these conventional watermarking algorithms demonstrate poor resistance to geometric attacks; hence, other models that can resist conventional attacks and geometric attacks effectively are necessary. Thus, the design and construction of a new medical image algorithm model based on perceptual hashing technology and neural network technology should be attempted to resolve the contradiction between the robustness and invisibility of medical image digital watermarking. Perceptual hashing mainly resolves the issue of conventional attacks and the neural network mainly resolves geometric attacks. The framework diagram is shown in Figure 3. The model process roughly uses the output vector of the hash algorithm as the input vector of the neural network, and finally obtains the output result. Perceptual hashing is a type of hashing algorithm, and its workflow has 7 main steps: 1) Reduce the size, reduce the picture to 8×8 size, a total of 64 pixels; 2) Simplify the color, that is, convert the reduced image to 64-level grayscale; 3) Calculate DCT. DCT is to decompose the frequency of the picture and gather it into a trapezoid shape. Here, a 32×32 DCT transform is used; 4) Reduce the DCT and keep the 8*8 matrix in the upper left corner, showing the lowest frequency in the picture; 5) Calculate the average of all 64 values; 6) To further reduce the DCT, set a 64-bit hash value of 0 or 1 according to the 8×8 DCT matrix, set the value greater than or equal to the average value of DCT to “1”, and set the value less than the average value of DCT to “0”; 7) Calculate the hash value. The neural network is a mathematical model or calculation model that imitates the structure and function of a biological neural network. It is calculated by connecting a large number of artificial neurons, mainly including an input layer, a hidden layer and an output layer.

The robustness and invisibility of digital watermark images can be studied from the following perspectives:

1) Regarding anti-conventional attacks, research is based on the extraction of perceptual hashing medical image features in the transform domain. It is used to study the human visual system, and by combining with perceptual hashing technology, establishes a transform domain perception hash algorithm model, and locates a vector that conforms to the human visual characteristic and is robust against conventional attacks.
2) Regarding anti-geometric attacks, the extraction of medical image features based on perceptual hashing and a neural network is studied. The Osirix DICOM image library and existing medical images are adopted to construct a medical image database that is attacked using nonlinear geometry. Then, the neural network model is designed to train the 2D and 3D medical images after nonlinear geometric attacks, and find the robust feature vectors against nonlinear geometric attacks, which are used as the features of designing robust watermarking algorithms for medical images against geometric attacks.
3) Research on methods for extracting robust perceptual hashing sequences from medical images based on perceptual hashing and neural networks.
4) Regarding research on how to embed large-capacity digital watermarks in medical images, perpetual hashing sequence feature vectors that counter conventional attacks and geometric attacks are used to generate a secret key by combining with the encrypted watermark to complete the embedding and extraction of a large-capacity watermark.

### Research on Gene Expression Profile Data Based on Low-Rank Sparse Representation

The emergence of gene expression profile data helps the understanding of the pathological process of cancer cells at the molecular level. Tens of thousands of varying genes in tissue samples can be detected by gene chips, and then the gene chip expression profile data can be analyzed and processed. Thus, tumors are classified so that patients can be treated effectively. However, gene expression profiles are characterized by high dimensionality, large noise, a small number of gene samples, missing data, data redundancy, and an unbalanced distribution of class samples. Thus, advanced data modeling methods must be used to extract the classification characteristics of samples effectively from tens of thousands of gene expression profiles. With the rapid development of artificial intelligence and machine learning in speech and machine vision in recent years, the use of machine learning methods, including deep learning, to explore gene expression profile data modeling methods is destined to be a development trend in the future.

Presently, research on gene expression profiles mainly covers the following: 1) the preprocessing of gene expression profile data, 2) extraction of gene expression profile data features, 3) selection of gene expression profile data features, and 4) clustering and classification research of gene expression profile data. Common gene feature selection methods are categorized into three types: the filter method, wrapper method, and embedded method (Bolón-Canedo et al., 2014). They can also be based on low-rank scoring, low-rank representation coefficient-based gene feature correlation measurement, and a two-step method based on robust principal component analysis (RPCA) (Partridge and Jabri, 2002) and the maximum margin criterion (MMC) for feature selection. RPCA, low-rank representation (Shu et al., 2017), and matrix completion (Cao et al., 2011; Zeng et al., 2017; Liu et al., 2020c; Ran et al., 2020; Zhao et al., 2020) are three main research areas for low-rank sparse theory. As the name implies, sparse representation refers to a linear combination of fewer basic signals to express most or all of the original signal. Among them, these basic signals are called atoms, which are selected from the over-complete dictionary; and the over-complete dictionary is gathered from atoms whose number exceeds the signal dimension. Therefore, it can be seen that any signal has different sparse representations under different atom groups. For example, a M×N matrix is used to represent the data set X , each row represents a sample, and each column represents an attribute of the sample. Generally speaking, the matrix is dense, that is, most elements are not 0. The meaning of sparse representation is to find a coefficient matrix A(K×N) and a dictionary matrix B(M×K) , so that B×A restores X as much as possible, and A is as sparse as possible. A is the sparse representation of X .

**FIGURE 4:**
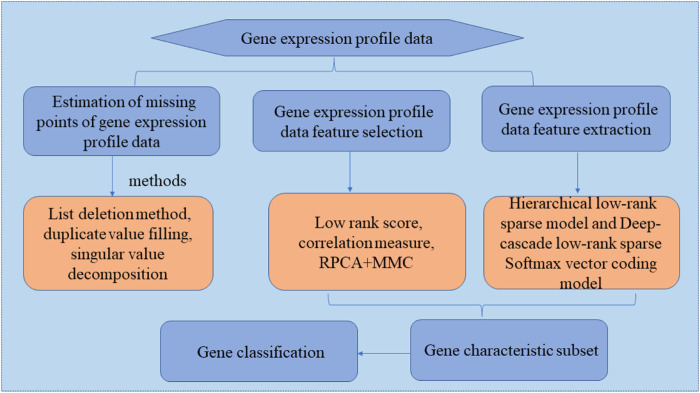
Research procedure for gene database analysis based on low-rank sparse representation.

Low-rank sparse representation models have been applied in many fields (Cheng et al., 2016; Chen et al., 2017; Zhang et al., 2017; Brbic and Kopriva, 2018; Chen et al., 2018; Xie et al., 2018; Yuanyuan et al., 2018; Zeng et al., 2018; Ding et al., 2019; Shen et al., 2019; Zhang et al., 2019; Li et al., 2020; Wu and Yu, 2021), which demonstrate high superiority, particularly in terms of dimensionality reduction and subspace segmentation. Considering existing analysis methods, introduce a low-rank sparse representation model for gene expression profile data analysis, several new methods for feature selection and feature extraction of gene expression profile data based on low-rank sparse representation models are explored, and they are applied to gene expression profile clustering and classification. As shown in Figure 4, this section mainly uses the following process to study gene expression profile data based on low-rank sparse representation analysis. In typical cases, the following three specific research areas are mainly involved when studying gene expression profile data.

1) Estimation of missing points in gene expression profile data.

In recent years, missing point estimation methods have included the following: 1) list deletion method; 2) duplicate value filling; 3) average value substitution method; and 4) the use of statistical methods for estimation, such as *K*-nearest neighbor (KNN) (Olga et al., 2001), singular value decomposition, and local least squares.

2) Feature selection for gene expression profile data.

Feature selection is a major prerequisite for the classification and clustering of gene expression profile data (Lu and Zhao, 2019; Zou et al., 2020; Qi et al., 2021a; Zulfiqar et al., 2021). Three common gene feature selection methods exist: the filter method, wrapper method, and embedded method. And Low-rank scoring, gene feature correlation measurement based on a low-rank representation coefficient, and a two-step method based on RPCA and MMC can also be used to select features. To overcome the shortcomings of traditional low-rank representation models, feature selection introduces manifold regularization constraints and class-label information constraints, sets up a manifold regularized low-rank representation model and a class-label constrained low-rank representation model, and solve the low-rank representation coefficient matrix in the two models. On this basis, two different low-rank graphs are set up, the low-rank graphs are used to score each gene feature, and a set of optimal gene feature subsets is selected according to the score.

3) Gene expression profile data feature extraction.

Common feature extraction methods can be divided into linear and nonlinear transformations. Typical linear feature extraction algorithms include sparse principal component analysis (PCA) (Min et al., 2018; Islam et al., 2020), independent component analysis (Moysés et al., 2017), and LDA. Nonlinear transformation methods primarily include neural networks, kernel methods (Qi et al., 2021b), manifold learning (Shen et al., 2017), sparse representation (Min et al., 2017), and matrix factorization methods (Wang et al., 2017; Yang et al., 2017; Yang and Hu, 2017; McCall et al., 2019). With the continuous development of machine learning and data mining, new feature extraction methods continue to arise. For example, PCA, FA, and ICA are three characteristic methods commonly used in gene expression profile data mining.

Gene expression profile data analysis has attracted widespread attention from scholars, and a series of gene expression profile analysis methods have been proposed. Classic methods such as PCA, LDA, KNN, decision-making tree method, ensemble learning, SVM, extreme learning machine, neural network, sparse representation, and gene bi-clustering method based on qualitative/quantitative measurement have been widely applied to the classification and clustering of gene expression profile data. Meanwhile, these technologies can provide techniques and comparisons for low-rank sparse representation methods. The core of the low-rank sparse representation method is low-rank sparse modeling theory. As an effective tool for large-scale data analysis, this theory has made great progress in recent years. Additionally, it has been widely used in subspace segmentation, image processing and recognition, machine vision, system modeling and control, and other large-scale data analysis.

## Conclusion

Therefore, it has become an inevitable trend to apply low-rank sparse representation models to study them. Low-rank sparse representation models have been applied in multiple fields, particularly in dimensionality reduction and subspace segmentation. For example, in feature extraction, traditional graph-based learning algorithm feature extraction methods are constrained using a graph construction method, and the effectiveness of the extracted feature vectors is reduced. By contrast, low-rank graphs have better local and global data description capabilities. A dimensionality reduction method based on low-rank graphs is a more effective feature extraction method. Moreover, with the advancement of biological sequencing technology, scientists have been able to observe the gene expression of cancer cells at the single-cell level, and discovered that the heterogeneity of cancer tissue far exceeds previous estimates. However, so far, low-rank sparse representation models are rarely used for gene data analysis. Therefore, this article introduces low-rank sparse representation models for gene expression profile data analysis based on existing analysis methods. Discuss new methods for feature selection and feature extraction of gene expression profile data based on low-rank sparse representation model, and use it for gene expression profile clustering and classification.

At the same time, with the advancement of biological sequencing technology, scientists have been able to observe the gene expression of cancer cells at the single-cell level, and found that the heterogeneity of cancer tissues far exceeds previous estimates. The observation samples of potential strongly heterogeneous data are likely to be in multiple feature subspaces. Each subspace is composed of the same set of molecular features that represent the same cancer class (subtype), and samples from different subspaces belong to different cancer class (subtype). However, many unsupervised methods proposed before cannot distinguish different feature subspaces, so errors or even invalid results may occur when these methods are used for cancer molecular feature mining. After research, it is found that the low-rank representation learning algorithm can accurately identify the inherent sample “cluster” structure or grouping information in heterogeneous data. The algorithm assumes that the sample subspace exists, and samples in the same subspace can characterize each other. Samples in different subspaces cannot characterize each other. Moreover, the effectiveness of this algorithm has been widely recognized in the field of image processing, and it also provides us with new ideas and new directions for establishing accurate models for mining cancer molecular characteristics.

## Pending Issues and Prospects

Gene expression profile data analysis has attracted widespread attention from scholars at home and abroad. Not only have they proposed a series of gene expression profile analysis methods, but they also developed a variety of gene software based on gene public databases, such as EASE network platform, pathway analysis software Gen-MAPP2 and the development of the domestically developed pathway analysis platform KOBAS, the development of these software provides a basis for the subsequent further research on gene expression profiles.

This article mainly uses low-rank sparse modeling theory to analyze experimental data. As one of the effective tools for large-scale data analysis, this theory has been widely used in different aspects in recent years. For example, sparse representation has been applied to the field of pattern recognition and has yielded fruitful results. The low-rank sparse representation model based on sparse representation has also become a research focus in machine vision, machine learning, and image processing, and has been applied successfully in video image processing, target recognition, task learning, bioinformatics (Ding et al., 2020; Hong et al., 2020; Hu et al., 2020; Lu et al., 2020; Hu et al., 2021a; Hu et al., 2021b; Wang et al., 2021b), and recommendation systems (Wei et al., 2014; Wei et al., 2017a; Wei et al., 2017b). However, further attention should be paid to low-rank representation learning. In specific applications, LRR generally uses original data as a dictionary, which requires a sufficient number of observed data samples, and only part of the data in the dictionary can be damaged. In real-world scenarios, the aforementioned assumptions may not be tenable; hence, LatLRR can be considered, and a dictionary can be constructed using observed and unobserved data.

At the same time, sparse representation also has important clinical significance. For example, data released by the National Cancer Center reveal that there are approximately 4.29 million new cancer patients in China every year, which accounts for 20% of new cases globally, and deaths have reached 2.81 million. Approximately 10,000 patients are diagnosed with cancer in China every day, that is, one patient every 7 min. Therefore, the prevention and treatment of cancers are not optimistic. It is expected that the incidence of cancers will continue to rise in the next one or two decades. The high incidence of cancer cases has resulted in severe challenges to domestic economic development and residents’ healthy life. How to prevent and treat cancer effectively has become a topic of great interest worldwide. With the advancement of high-throughput technology, biomedicine is rapidly stepping into the era of big data. Omics data represented by gene expression profiles have demonstrated particular leaps. The emergence of gene expression profile data helps people to understand the pathological process of cancer cells at the molecular level. Thousands of genes in tissue samples can be detected by gene chips, and then the tumor can be classified by analyzing and processing the gene chip expression profile data so that patients can be treated effectively. However, because of the characteristics of gene expression profile data, there are still many problems in the research field. With the rapid development of artificial intelligence and machine learning in the field of speech and machine vision, in the next few years, artificial intelligence and machine learning will play an increasingly important role in genetic biology, genomic medicine and precision medicine, especially deep learning. The rapid development has attracted widespread attention from researchers in the biomedical field, so it has become an inevitable trend to use low-rank sparse representation models to study them. An extensive application of sparse representation in bioinformatics helps to address the problem that some unsupervised algorithms cannot distinguish different feature subspaces of cancer molecules. Moreover, it is expected that, in the near future, it can provide technological references for the prevention and treatment of critical illness, and the research and development of new drugs.

However, sparse representation in bioinformatics still has varying degrees of limitations. For example: 1) Constructing a more flexible sparse representation model. In the existing sparse representation model, there is an objective function and a constraint function, the objective function is generally to minimize the energy of the noise under the assumption that the observation signal has a linear model form and contains Gaussian white noise, constraint function generally refers to sparse constraint term. On the one hand, this objective function treats the sparse components equally; on the other hand, it ignores the existence of other goals in different applications, because if you look at it from the standpoint of representation alone, it does not necessarily require the sparsest solution to be unique or the sparsest solution is not the most ideal. Therefore, it is necessary to construct a sparse representation model with multiple targets and variable regular parameters to meet the characteristics and needs of more application problems. 2) When determining the regular parameter λ and the parameter k representing the degree of sparseness for the model, a manual pre-determined method is generally used to assign values to the two hyperparameters. After determining its value, perform the solution, and then compare the solution result with the target demand. If it does not meet the requirements, then adjust the parameters. This inevitably results in non-adaptability or non-automation of the solution process, and also limits the application of sparse representation methods in some fields that require a high degree of automation. Therefore, it is necessary to study the adaptive solution of sparse representation model, and construct the functional relationship between hyperparameters and observation signals and sparse vectors. 3) At present, the application scope of sparse representation is mainly limited to the field of natural signals. The application prospects in the field of unnatural data signals are still unclear. According to the characteristics of sparse representation in various fields, the application types of sparse representation can be divided into reconstruction based Applications and classification-based applications. Reconstruction-based applications mainly include image denoising, image signal reconstruction, audio signal recovery, compressed sensing, SAR imaging, etc. The common point of this category of applications is that the characteristics of the target signal need to be obtained first, and the sparse vector is constructed using the characteristics. The mathematical model in the sparse representation theory is then used to solve the problem to achieve the effect of reconstructing the original signal within the allowable error range. Classification-based applications mainly include face recognition, target tracking, text detection, blind source separation, etc. Classification-based applications all construct sparse feature vectors by extracting feature information from objects. These feature vectors are strongly distinguishable and can differentiate different types of signals, and then according to the optimization method of sparse representation, determine the distance between the target signal and these feature vectors, and when a certain threshold is met, it is determined to belong to the category to achieve the effect of pattern recognition and classification. Therefore, sparse representation has some limitations in the application of bioinformatics, which requires further research and discussion by scholars.

At the same time, sparse representation provides a powerful means in blind source separation technology, because blind source separation technology is to solve the unknown input and unknown transmission channel and output the known signal processing technology. The sparse representation technology reduces the complexity of the algorithm by separating the estimation process of the mixing matrix and the estimation process of the source signal, and improves the accuracy of the source signal separation. Therefore, sparse representation has become a popular method in the current blind source separation problem.
